# β-Cyclodextrin Polymer-Based Fluorescence Enhancement Strategy via Host–Guest Interaction for Sensitive Assay of SARS-CoV-2

**DOI:** 10.3390/ijms24087174

**Published:** 2023-04-12

**Authors:** Shanshan Gao, Gege Yang, Xiaohui Zhang, Rui Shi, Rongrong Chen, Xin Zhang, Yuancheng Peng, Hua Yang, Ying Lu, Chunxia Song

**Affiliations:** 1 Department of Applied Chemistry, School of Science, Key Laboratory of Agricultural Sensors, Ministry of Agriculture and Rural Affairs, Anhui Agricultural University, Hefei 230036, China; 2 State Key Laboratory of Tea Plant Biology and Utilization, Hefei 230036, China; 3 School of Life Science, Anhui Agricultural University, Hefei 230036, China

**Keywords:** DNA aptamer, host–guest interaction, fluorescence enhancement, SARS-CoV-2, β-cyclodextrin polymer

## Abstract

Nucleocapsid protein (N protein) is an appropriate target for early determination of viral antigen-based severe acute respiratory syndrome coronavirus 2 (SARS-CoV-2). We have found that β-cyclodextrin polymer (β-CDP) has shown a significant fluorescence enhancement effect for fluorophore pyrene via host–guest interaction. Herein, we developed a sensitive and selective N protein-sensing method that combined the host–guest interaction fluorescence enhancement strategy with high recognition of aptamer. The DNA aptamer of N protein modified with pyrene at its 3′ terminal was designed as the sensing probe. The added exonuclease I (Exo I) could digest the probe, and the obtained free pyrene as a guest could easily enter into the hydrophobic cavity of host β-CDP, thus inducing outstanding luminescent enhancement. While in the presence of N protein, the probe could combine with it to form a complex owing to the high affinity between the aptamer and the target, which prevented the digestion of Exo I. The steric hindrance of the complex prevented pyrene from entering the cavity of β-CDP, resulting in a tiny fluorescence change. N protein has been selectively analyzed with a low detection limit (11.27 nM) through the detection of the fluorescence intensity. Moreover, the sensing of spiked N protein from human serum and throat swabs samples of three volunteers has been achieved. These results indicated that our proposed method has broad application prospects for early diagnosis of coronavirus disease 2019.

## 1. Introduction

Ever since December 2019, severe acute respiratory syndrome coronavirus 2 (SARS-CoV-2) has spilled over the world and has posed an unprecedented threat to public health worldwide [1,2,3]. People infected may experience systemic toxicity-like symptoms as well as respiratory symptoms, called coronavirus disease 2019 (COVID-19) [4]. Up to December 2022, the World Health Organization has reported more than 6.4 hundred million infections and 6.6 million deaths worldwide. As SARS-CoV-2 is constantly mutating and extremely infectious, tracking and isolating the contacts effectively will be beneficial to reduce the overall scale of the epidemic and take it under control over a longer period of time [5]. Therefore, there is an urgent need for the development of sensitive and effective methods for early diagnosis of COVID-19.

The current diagnosis for assessing SARS-CoV-2 infection mainly depends on nucleic acid-based detection (genetic material ribonucleic acid, RNA) and serological test of antibodies [6,7,8]. Nucleic acid testing requires a variety of expensive reagents, specialized equipment, and skilled technicians. More seriously, the instability of viral RNAs may lead to false-negative results owing to improper treatment or preservation during transport and storage [9,10,11,12]. By contrast, antigen tests could detect virus proteins that appear at the beginning of symptoms. SARS-CoV-2 contains nucleocapsid, spike, membrane, and envelope protein [13]. Nucleocapsid protein (N protein) is a kind of highly immunogenic protein which is expressed in large quantities during infection [14]. It could intertwine with the viral genomic RNA to form a nucleocapsid, which plays a vital role in the composite of viral RNA. Li et al. [15] have detected SARS-CoV-2 N protein in the serum of PCR-positive patients with a sensitivity of 92% and specificity of 97% before the antibody appearance, which shortens the window of serological diagnosis and has a high diagnostic value for patients in the early stages of infection. In another report, Li et al. [16] established microfluidic magneto immunosensors for rapid, highly sensitive, and quantitative measurements of SARS-CoV-2 N protein in serum samples and adapted the assay onto a handheld smartphone-based diagnostic device. These results proved that quantitative measurements of SARS-CoV-2 coronavirus antigens in serum/plasma are beneficial to accurate and early diagnosis of COVID-19 and can provide a way to supplement the gold-standard nucleic-acid detection by filling the test gaps. Furthermore, the quantification of the N protein is superior to that of the spike protein because it can avoid the problems related to the mutation of the latter, resulting in more reliable detection [17].

Besides immunodiagnosis using antigen-antibody reaction, developing novel strategies that are based on other high-affinity synthetic and more stable recognition receptors in the identification for building highly-selective and sensitive detection is quite beneficial to the war against COVID-19. DNA aptamers could recognize targets with high affinity in the form of antibodies [18]. As a kind of deoxyribonucleic acid sequence, they possess the advantages of easy synthesis and chemical modification, high affinity and stability, convenient operation, etc. Aptamers have acted as recognition receptors in the monitoring of viruses, including SARS [19], Ebola [20], and influenza [21]. Luo group [22] screened N protein aptamer with high affinity (the minimum K_d_ value was 0.49 ± 0.05 nM) in 2020. It has been successfully applied as a recognition element for the ultra-sensitive assay of COVID-19 in an electrochemical aptasensor by our group [17] and holds a promising prospect in many other analytical technologies.

**Figure 1:**
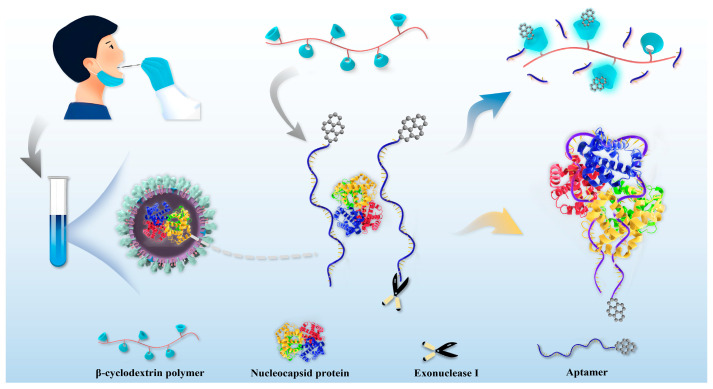
Schematic design of aptamer-based sensitive assay of SARS-CoV-2 N protein using β-cyclodextrin polymer to enhance pyrene fluorescence.

In this paper, we designed a fluorescence method for detecting the N protein of SARS-CoV-2 that combined the specific recognition capability of aptamer with the outstanding fluorescence enhancement of cyclodextrin polymers (β-CDP). Cyclodextrin polymer that constitutes monomer cyclodextrin through polymerization, graft copolymerization, or molecular cross-linking not only retains the characteristics of highly specific host–guest recognition of monomer cyclodextrin but also exhibits some advantages [23]. Our previous work proved that compared to cyclodextrin monomer, β-CDP holds a stronger fluorescence enhancement effect to pyrene besides retention of the highly specific host–guest identification characteristics [24,25]. Figure 1 showed that N protein aptamer modified by the fluorophore pyrene at its 3′-terminal could be hydrolyzed by exonuclease I (Exo I) to produce pyrene attached to a single nucleotide. Pyrene easily entered into the β-CDP cavity and showed obvious fluorescence enhancement. The aptamer could combine with the added N protein and turn into a complex, which prevented the hydrolysis of Exo I. The pyrene stilled attached to the intact aptamer and could not interact with β-CDP because of steric hindrance, thus affecting the fluorescence enhancement. The fluorescence emission increased with the decrease in N protein concentration. Meanwhile, combining the high affinity/specificity N protein aptamer with the outstanding fluorescence enhancement capability of β-CDP, a sensitive and selective strategy for early detection of SARS-CoV-2 could be achieved by measuring the fluorescent signal.

## 2. Results and Discussion

### 2.1. The Fluorescence Enhancement of β-Cyclodextrin Polymer

**Figure 2:**
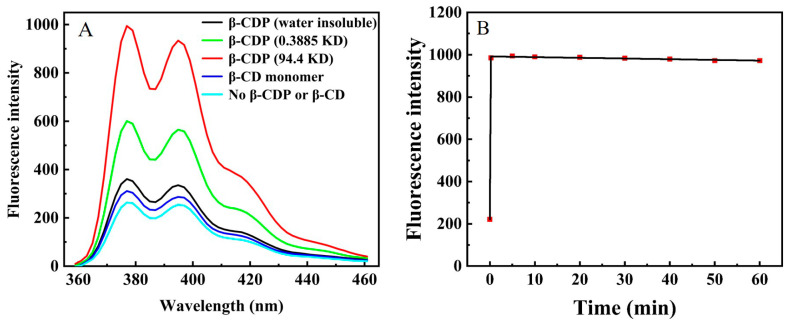
(**A**) Fluorescence emission spectra in addition to β-CDP or β-CD monomer. (**B**) Fluorescence intensity of pyrene in addition to β-CDP vs. time. The concentration of β-CDP was 1.5 mg/mL. The concentration of pyrene labeled aptamer was 400 nM. The maximum emission wavelength was set at 376 nm.

The effective fluorescence enhancement ability of β-CDP could guarantee the sensitivity of our proposed method. As shown in Figure 2A, the fluorescence enhancement of pyrene in addition to β-CD monomer or water-insoluble β-CDP was weak. While water-soluble β-CDP (the molecular weight was 94.4 KD and 0.3885 KD) could dramatically enhance the pyrene fluorescence. 94.4 KD β-CDP could enhance the pyrene fluorescence by about four times, and the enhanced fluorescence could be kept stable for at least 60 min (Figure 2B). These results demonstrated the efficient fluorescence stability and enhancement capability of 94.4 KD β-CDP, which was applied in subsequent experiments.

**Figure 3:**
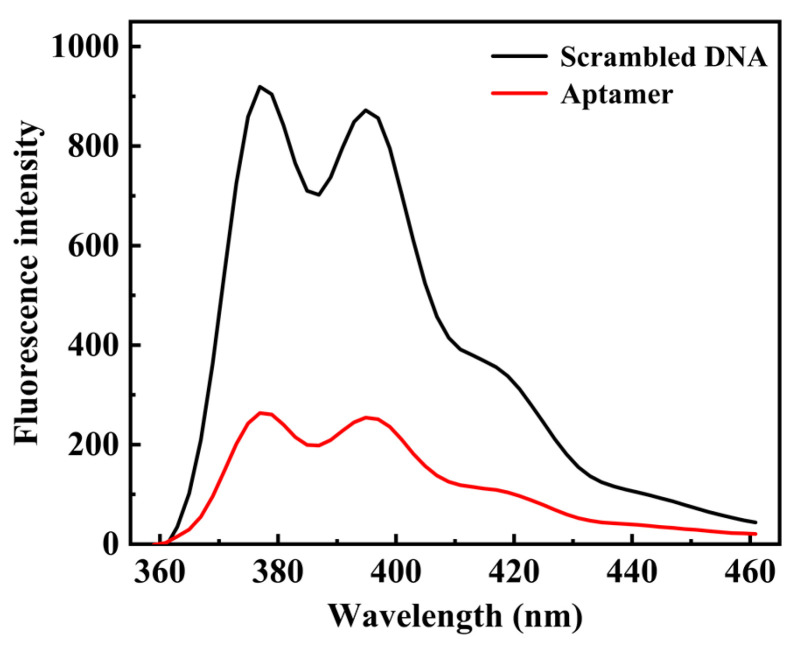
Fluorescence emission spectra in addition to pyrene-labeled scrambled DNA sequence and N protein aptamer. The concentration of β-CDP was 1.5 mg/mL. The concentration of N protein was 600 nM. The concentration of pyrene-labeled aptamer or pyrene-labeled scrambled DNA sequence was set at 400 nM. The maximum emission wavelength was set at 376 nm.

In order to exclude the nonspecific binding between DNA and N protein, pyrene-labeled scrambled DNA sequence or N protein aptamer was added to the system containing the same content of N protein. As shown in Figure 3, compared with scrambled DNA, the fluorescence of the system added with N protein aptamer decreased significantly, indicating that N protein can only be recognized and specifically bounded by N protein aptamer.

### 2.2. The Performance of N Protein Aptamer

**Figure 4:**
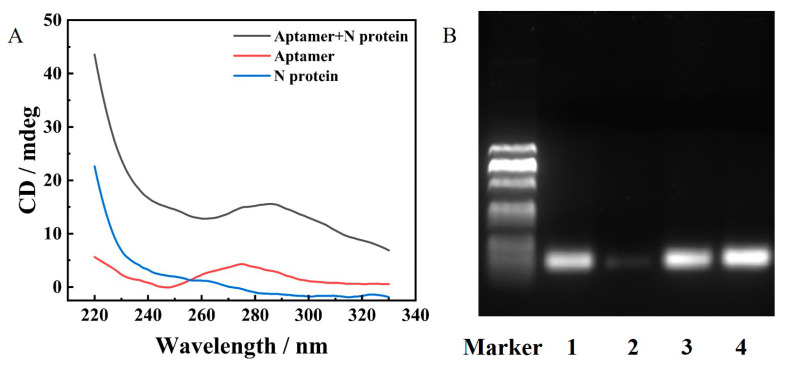
(**A**) CD spectra of aptamer after incubation with N protein at 37 °C for 30 min (black curve), aptamer (red curve), and N protein alone (blue curve). The concentration of N protein and aptamer was equal to 1 μM. (**B**) Agarose gel electrophoresis. The concentration of N protein, aptamer, and Exo I was 600 nM, 400 nM, and 240 U/mL, respectively.

To confirm the high affinity of N protein aptamer, we measured the circular dichroism (CD) spectra of the system that contained aptamer, N protein, and aptamer after incubation with N protein for 30 min at 37 °C, respectively. As shown in Figure 4A, the 280 nm peak value of the system that aptamer incubated with N protein was significantly increased compared to that of aptamer and N protein alone (at 280 nm, nearly zero). The change suggested that the aptamer could successfully incorporate with N protein after incubation to form a hybrid and confirmed the high affinity of N protein aptamer.

Furthermore, gel electrophoresis was used to explore whether the combination of N protein and aptamer could effectively protect aptamer from digestion by Exo I. As shown in Figure 4B, lane 1 showed single-stranded aptamer; lane 2 represented aptamer incubated with Exo I at 37 °C for 30 min and then heated at 80 °C for 20 min; lane 3 was aptamer incubated with N protein and the addition of Exo I at 37 °C for 30 min and then was heated at 80 °C for 20 min, and lane 4 showed the aptamer after incubation with N protein at 37 °C for 30 min and then was heated at 80 °C for 20 min. Lane 1 and lane 4 showed bright bands, which proved that the aptamer was not decomposed without Exo I, so the pyrene was still attached to the aptamer. As for lane 2, there was no band in the absence of N protein, which proved that aptamer had been digested by Exo I in the absence of N protein, followed by the release of pyrene. In the case of the N protein presented (lane 3), there was a bright band, which indicated that the incorporation of N protein and aptamer could protect the aptamer from Exo I decomposition.

### 2.3. Optimization Experiment

In this study, some factors, such as the concentration of Tris-HCl buffer pH, the concentrations of β-CDP, the concentration of Exo I, and the concentration of aptamer would affect the performance of the detection method; thus, the effects of these factors were investigated in order to obtain high effective analysis performance for N protein assay. (F_0_–F)/F_0_ ratio was used as a standard to select the optimum condition. F_0_ and F are the fluorescence of the system at 376 nm in the case of blank and target N protein (200 nM), respectively.

**Figure 5:**
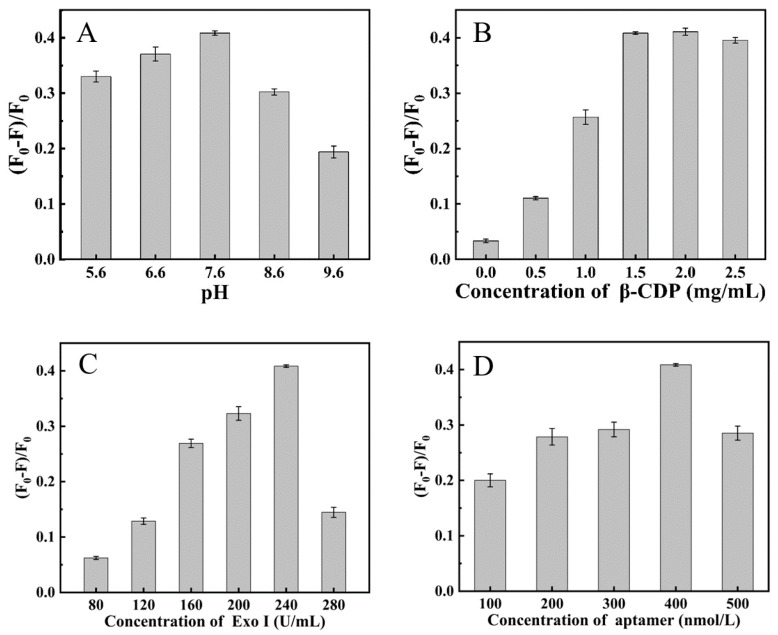
(**A**) Optimization of pH value. The activity of Exo I was 240 U/mL. The concentration of N protein, aptamer, and β-CDP was 200 nM, 400 nM, and 1.5 mg/mL, respectively. (**B**) (F_0_–F)/F_0_ vs. the centration of β-CDP. The activity of Exo I was 240 U/mL. The concentration of N protein and aptamer was 200 nM and 400 nM, respectively. (**C**) (F_0_–F)/F_0_ vs. the activity of Exo I. The concentration of aptamer was 400 nM. The concentration of N protein was 200 nM. The concentration of β-CDP was 1.5 mg/mL. (**D**) (F_0_–F)/F_0_ vs. the concentration of aptamer. The concentration of Exo I and N protein were 240 U/mL and 200 nM, respectively. The concentration of β-CDP was 1.5 mg/mL. Error bars indicated the standard deviations of three experiments.

We investigated the effect of Tris-HCl buffer pH and found that the pH value has a great influence on the experimental system (Figure 5A). The pH value affected the cleavage ability of Exo I to N protein aptamers, and the system showed the best performance when the buffer pH value of the system was 7.6. We studied the changes in fluorescence intensity of the system under different concentrations of β-CDP. Figure 5B showed that (F_0_–F)/F_0_ increased greatly with the addition of β-CDP and then kept constant after the concentration of 1.5 mg/mL, which was used for further research. As shown in Figure 5C, (F_0_–F)/F_0_ achieved the maximum when the concentration of Exo I was 240 U/mL. As shown in Figure 5D, with the increase in the aptamer concentration, (F_0_–F)/F_0_ gradually increased and then reached the maximum when the concentration of aptamer was 400 nM. When the aptamer concentration was greater than 400 nM, the extra aptamer was cut by Exo I, which resulted in the increase of the pyrene concentration and the fluorescence intensity F_0_ of the system, while F_0_−F (which was positively related to N protein) remained unchanged, so (F_0_–F)/F_0_ became smaller. As for the Exo I, the extra Exo I could hydrolyze the complex of an aptamer that combines with N protein, which resulted in the increase of F (the system with N protein included), while F_0_ (the system without N protein included) remains unchanged, so (F_0_–F)/F_0_ becomes smaller. So pH 7.6, 1.5 mg/mLβ-CDP, 400 nM aptamer, and 240 U/mL Exo I were used for further study in this work.

### 2.4. Analytical Performance

**Figure 6:**
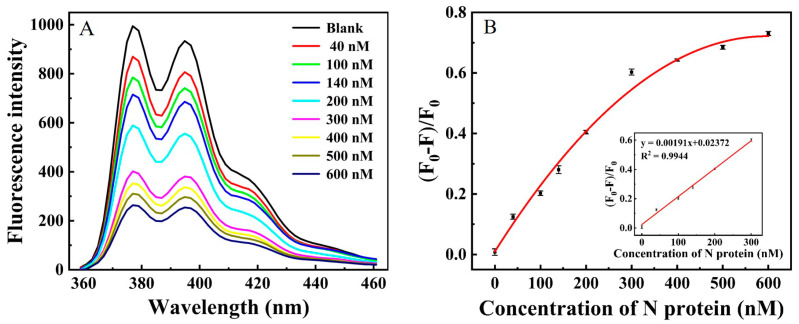
(**A**) Fluorescence emission spectra of the system under the concentration of N protein from 0 to 600 nM. (**B**) The linear relationship between the concentration of N protein (the concentration from left to right was 0, 40, 100, 140, 200, and 300 nM, respectively) and (F_0_–F)/F_0_ (S/N = 3). The concentration of aptamer, Exo I, and β-CDP was 400 nM, 240 U/mL, and 1.5 mg/mL, respectively. The excitation wavelength was set at 345 nm, and the maximum emission wavelength was set at 376 nm.

We set out to detect a concentration gradient experiment of N protein under optimal conditions to validate the performance of this strategy in quantitative detection. Figure 6A displayed that the fluorescence emission was remarkably decreased with the increase in N protein concentration. In this study, (F_0_–F)/F_0_ increased linearly with the concentration of N protein. The linear range was 50–300 nM (R^2^ = 0.9944) with a low detection limit of 11.27 nM (S/N = 3), which reached the sensitivity of some reported SARS-CoV-2 assays that also depended on viral proteins detection [16]. Its high sensitivity was attributed to the outstanding fluorescence enhancement ability of β-CDP. In addition, β-CDP that we used in this work was a little electronegative [24]. The mutually repulsive force between the negatively charged aptamer and β-CDP could guarantee the low background; thus, it was also conducive to the high sensitivity.

### 2.5. Selectivity

**Figure 7:**
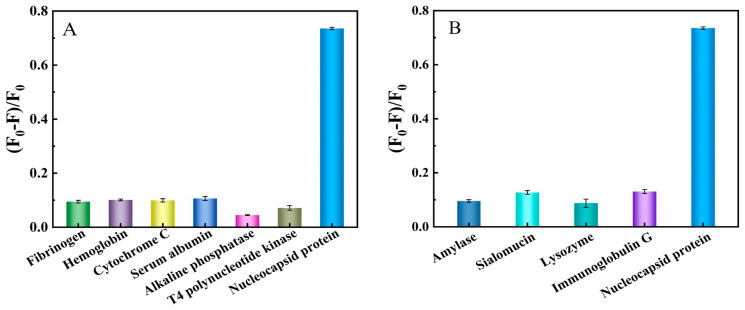
(F_0_–F)/F_0_ value of potential interferences in human blood samples (**A**) and throat swabs (**B**). The concentration of potential interferences or N protein was all set at 600 nM.

To verify the selectivity of our proposed method, we selected six commonly found proteins (fibrinogen, hemoglobin, cytochrome C, serum albumin, alkaline phosphatase, T4 polynucleotide kinase) in blood serum as potential interferences. Figure 7A shows (F_0_–F)/F_0_ of the system while coexistents or N protein were added with a final concentration of 600 nM. (F_0_–F)/F_0_ of the system was 0.735 as for N protein, in the case of fibrinogen, hemoglobin, cytochrome C, serum albumin, alkaline phosphatase or T4 polynucleotide kinase, (F_0_–F)/F_0_ of the system accounted for only 0.095, 0.101, 0.100, 0.107, 0.045, and 0.072, respectively.

We also selected four proteins (amylase, sialomucin, lysozyme, immunoglobulin G) usually discovered in saliva as potential interferences for selective experiments. Figure 7B showed (F_0_–F)/F_0_ of the system when potential interferences or N protein were added into the system with a final concentration of 600 nM. Compared with (F_0_–F)/F_0_ by more than 0.7 in addition to N protein, (F_0_–F)/F_0_ of the system in the case of amylase, sialomucin, lysozyme, immunoglobulin G accounted for only 0.096, 0.127, 0.088, and 0.131, respectively. These results proved the high selectivity of our proposed method and showed the application prospect in serum and saliva sample detection.

### 2.6. N Protein Analysis in Clinical Blood and Throat Swabs Samples

**Figure 8:**
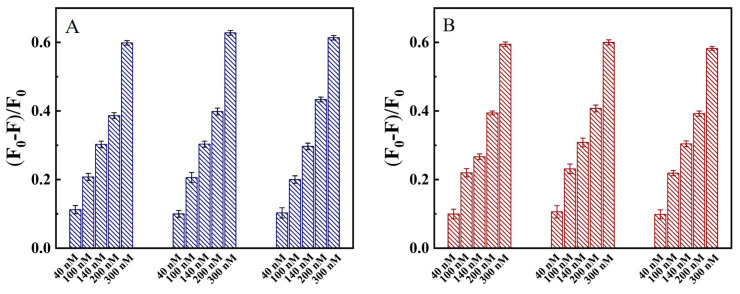
(**A**) (F_0_–F)/F_0_ value at various concentrations of N protein spiked to the human serum sample. (**B**) (F_0_–F)/F_0_ value at various concentrations of N protein spiked to the throat swabs sample.

Blood and throat swab samples were taken an hour later after dining from three healthy volunteers, and the basic clinical features of the three volunteers are seen in Table 1. The blood was centrifuged at 4000 rpm/s for 5 min to obtain supernatant as serum samples. The pharyngeal swabs were soaked in 2 mL of Tris-HCl buffer. Various concentrations of N protein (the final concentration was 40, 100, 140, 200, and 300 nM) were spiked into the throat swabs and 10% serum samples, and then the detection was carried out. Figure 8 shows that (F_0_–F)/F_0_ of throat swabs and 10% serum samples increased gradually with the concentration of spiked N protein.

### 2.7. The Recovery of N Protein in Clinical Blood and Swabs Sample

We used statistical methods to obtain the average recovery rate of N protein from blood and throat swab samples in order to evaluate the accuracy of this method in detecting real samples (Table 2). The amounts of 40 nM, 100 nM, 140 nM, 200 nM, and 300 nM of N protein were added to the 10% serum and throat swab samples. Each concentration was set in three groups in parallel, and the real sample was not added with N protein for comparison. Lastly, the ratio of concentration obtained by this method to the spiked concentration was used to calculate the recovery rate.

## 3. Materials and Methods

### 3.1. Materials

N protein was manufactured by Sino Biological Inc. (Beijing, China). Epichlorohydrin, sodium hydroxide, isopropanol, tris hydroxymethyl aminomethane, and toluene were provided by China National Pharmaceutical Group Co., Ltd. (Beijing, China). DNAs were bought from TaKaRa Bio. Inc. (Dalian, China). Serum albumin was brought from Shandong Sikejie Biotechnology Co., Ltd. (Binzhou, China) β-cyclodextrin was supplied by Sigma–Aldrich Co., Ltd. (Shanghai, China). T4 polynucleotide kinase, lysozyme, Exo I, and 10 × Exo I buffer (67 mM MgCl_2_, 10 mM dithiothreitol, pH = 9.5, 670 mM glycine-KOH) were provided by Sangon Biotech Co. Ltd. (Shanghai, China). Fibrinogen, immunoglobulin G, hemoglobin, amylase, and cytochrome C were supplied by Beijing Soleibo Technology Co., Ltd. (Beijing, China). Alkaline phosphatase was purchased from Shanghai Biyuantian Biological Co., Ltd. (Shanghai, China). Sialomucin was obtained from Shanghai Yuanye Bio-Technology Co., Ltd. (Shanghai, China). Two electroneutral β-CDP (one of the molecular weights was 0.3885 KD, and the other was water insoluble) were bought from Shandong Zhiyuan Biotechnology Co., Ltd. (Binzhou, China).

### 3.2. DNA Sequences Used in this Work

All the information about DNA sequences used in this work were could be seen in Table 3.

### 3.3. Preparation of β-Cyclodextrin Polymer (β-CDP)

The synthesis of 94.4 KD β-CDP was the same as in our previous work [15]. An amount of 5 g (4.4 mmol) β-CD was dissolved in 15 mL 15% NaOH aqueous solution and stirred for 2 h at 35 °C, and then 1 mL (4.4 mmol) methylbenzene was added. Later, 4.4 mmol epichlorhydrin was added drop by drop and stirred for 3 h at 35 °C. An amount of 100 mL isopropanol was added to the mixture, and 6 mol/L HCl was used to adjust the pH value of the flocculating constituent to 7.0. Then the mixture was poured into a dialysis tube (MWCO 5000~8000) and dialyzed in pure water to remove the monomer and small molecules. Finally, the dialysate in the dialysis tube was dried at −60 °C through a vacuum dryer. Thereby, the white water soluble β-CDP was synthesized. The average particle size distribution of β-CDP was close to 207 μm due to the high solution concentration and cross-linking. The Zeta potential diagram of β-CDP was −15 mV, indicating that the polymer was weakly electronegative. The characterization details of β-cyclodextrin polymer could be seen in our previous work [26].

### 3.4. N Protein Detection

Exo I buffer, 12 μL of 5 μM aptamer solution, and various concentrations of N protein were added into centrifuge tubes and were incubated in a dry bath for 30 min at 37 °C. Afterward, Exo I was added into the centrifuge tubes and continued with 30 min incubation at 37 °C. Subsequently, after heating at 80 °C for 20 min in order to deactivate Exo I, 15 μL of 15 mg/mL β-CDP was added to the centrifuge tubes. At last, the Agilent G9800A fluorescence spectrophotometer was used for the detection of fluorescence signals, with the excitation wavelength set at 345 nm.

### 3.5. Investigation of Assay Selectivity

Control experiments were performed with six coexistent proteins fibrinogen, hemoglobin, cytochrome C, serum albumin, alkaline phosphatase, and T4 polynucleotide kinase in blood serum as potential interferences for the evaluation of the selectivity of our designed method. Four commonly found proteins, amylase, sialomucin, lysozyme, and immunoglobulin G in saliva, were also selected as potentially interfering substances. Different potential interfering substances or N protein with the same final concentration of 600 nM was added into the aptamer-contained buffer, followed by 30 min incubation at 37 °C, and then Exo I was added and continued for 30 min incubation at 37 °C. Subsequently, after heating at 80 °C for 20 min in order to deactivate Exo I, 15 μL of 15 mg/mL β-CDP was mixed into the mixture. Finally, the fluorescence signals with potential interfering substances or N protein were detected, respectively.

This study included one male and two female volunteers. For more than seven days in a row, their temperature was monitored and recorded as normal. Their specific clinical characteristics are described in Table 1.

### 3.6. Detection of N Protein in Clinical Blood and Swab Samples

The venous blood of three volunteers was taken and stored in EDTA-K2 anticoagulant tube. The blood samples were centrifuged at 4000 rpm/s to get the supernatant as serum. After adding different concentrations of N protein into 10% serum samples, the fluorescence signal was detected by this method.

Swallow swabs were collected one hour after the meal and put into 2 mL Tris-HCl buffer solution. N proteins with different concentrations (for example, 40 nM, 100 nM, 140 nM, 200 nM, and 300 nM) were added to the above throat swab samples. After that, the throat swab samples mixed with N protein were detected by the fluorescence method studied in this paper, and the blank throat swab samples without N protein were used as the control group.

## 4. Conclusions

Tracking and isolating the contacts effectively will be beneficial to reduce the overall scale of the epidemic and take it under control over a longer period of time, as SARS-CoV-2 is constantly mutating and extremely infectious [5]. So the development of sensitive and effective methods for early diagnosis of COVID-19 is in urgent need. Different from the current main diagnosis for assessing SARS-CoV-2 infection nucleic acid-based detection and serological test of antibodies [6,7,8], antigen tests could detect virus proteins that appear at the beginning of symptoms. N protein of SARS-CoV-2 is a kind of highly immunogenic protein and is expressed in large quantities during infection [14]. Previous research results [15] have proved that quantitative measurements of SARS-CoV-2 coronavirus antigens in serum/plasma are beneficial to accurate and early diagnosis of COVID-19 and can provide a way to supplement the gold standard nucleic acid detection by filling the test gaps.

We have constructed a fluorescence method for N protein monitoring that combined the host–guest interaction fluorescence enhancement strategy with high recognition of aptamer. The outstanding fluorescence enhancement during host–guest interaction between β-CDP/pyrene and the mutually repulsive force between the negatively charged aptamer and β-CDP guaranteed the low background and was conducive to the high sensitivity. Sensitive detection of N protein has been realized with the LOD as low as 11.27 nM with the linear range of 50–300 nM (R^2^ = 0.9944). This method exhibited significant selectivity against target among ten coexisting proteins (fibrinogen, hemoglobin, cytochrome C, serum albumin, alkaline phosphatase, T4 polynucleotide kinase in blood serum, and amylase, sialomucin, lysozyme, and immunoglobulin G usually discovered in saliva), attributed to the highly selective aptamer with high affinity. The more important thing is its successful application in the assay of spiked N protein of throat swabs and human serum samples, which proved that our method holds broad application prospect for sensitive detection of SARS-CoV-2 in human samples and early diagnosis of COVID-19.

## Figures and Tables

**Table 1:** Basic clinical characteristics of three volunteers.

Volunteer	Sex	Age	Height (cm)	Weight (kg)	Healthy	Nucleic Acid Test Results Within 48 h
1	Female	22	158	45	Yes	negative
2	Male	25	174	63	Yes	negative
3	Female	42	167	53	Yes	negative

**Table 2:** The recovery of N protein in clinical blood and swabs sample.

Spiked (nM)	Blood Samples					Throat Swabs				
	Recovery (%)				RSD (%)	Recovery (%)				RSD (%)
	1	2	3	Average (%)		1	2	3	Average (%)	
40	108.1	101.8	108.6	106.2	3.8	99.3	107.4	98.0	101.6	5.1
100	96.3	103.6	92.3	97.4	5.7	102.7	108.7	103.4	104.9	3.3
140	103.6	105.5	102.3	103.8	1.6	106.4	90.83	104.8	100.7	8.6
200	94.9	104.4	107.3	102.2	6.5	97.02	100.4	96.5	98.0	2.1
300	100.3	105.9	103.0	103.1	2.8	100.5	99.607	97.5	99.2	1.6

**Table 3:** DNA sequences used in this work.

Name	Sequence (5′ to 3′)
N protein-binding aptamer	5′-GCTGGATGTCGCTTACGACAATATTCCTTAGGGGCACCGCTACATTGACACATCCAGC-3′
Pyrene-labeled N protein binding aptamer	5′-SH-GCTGGATGTCGCTTACGACAATATTCCTTAGGGGCACCGCTACATTGACACATCCAGC-pyrene-3′

## Data Availability

Not applicable.

## References

[1] Wang Z., Morrissey J.J. (2022). Plasmonically enhanced ultrasensitive epitope-specific serologic assay for COVID-19. Anal. Chem..

[2] Zhong J., Rösch E.L., Viereck T., Schilling M., Ludwig F. (2021). Toward rapid and sensitive detection of sars-cov-2 with functionalized magnetic nanoparticles. ACS Sens..

[3] Wu F., Zhao S., Yu B., Chen Y.M., Wang W., Song Z.G., Hu Y., Tao Z.W., Tian J.H., Pei Y.Y. (2020). A new coronavirus associated with human respiratory disease in china. Nature.

[4] Zhu N., Zhang D., Wang W., Li X., Yang B., Song J., Zhao X., Huang B., Shi W., Lu R. (2020). A novel coronavirus from patients with pneumonia in china 2019. N. Engl. J. Med..

[5] Hellewell J., Abbott S., Gimma A., Bosse N.I., Jarvis C.I., Russell T.W., Munday J.D., Kucharski A.J., Edmunds W.J., Funk S. (2020). Feasibility of controlling COVID-19 outbreaks by isolation of cases and contacts. Lancet Glob. Health.

[6] Lukose J., Chidangil S., George S.D. (2021). Optical technologies for the detection of viruses like COVID-19: Progress and prospects. Biosens. Bioelectron..

[7] Cho S.Y., Jin X., Gong X. (2021). Antibody-free rapid detection of SARS-CoV-2 proteins using corona phase molecular recognition to accelerate development time. Anal. Chem..

[8] Udugama B., Kadhiresan P., Kozlowski H.N., Malekjahani A., Li M., Osborne V.Y.C., Chen H., Mubareka S., Gubbay J.B., Chan W.C.W. (2020). Diagnosing COVID-19: The disease and tools for detection. ACS Nano.

[9] Shi L., Wang L., Ma X., Fang X., Xiang L., Yi Y., Li J., Luo Z., Li G. (2021). Aptamer-functionalized nanochannels for one-step detection of SARS-CoV-2 in samples from COVID-19 patients. Anal. Chem..

[10] Eissa S., Zourob M. (2021). Development of a low-cost cotton-tipped electrochemical immunosensor for the detection of SARS-CoV-2. Anal. Chem..

[11] Wei C., Yang H., Wang S., Zhao J., Liu C., Gao L., Xia E., Lu Y., Tai Y., She G. (2018). Draft genome sequence of camellia sinensis var. sinensis provides insights into the evolution of the tea genome and tea quality. Proc. Natl. Acad. Sci. USA.

[12] Peng Y., Chen L., Li S., Zhang Y., Xu R., Liu Z., Liu W., Kong J., Huang X., Wang Y. (2018). BRI1 and BAK1 interact with G proteins and regulate sugar-responsive growth and development in Arabidopsis. Nat. Commun..

[13] Kumar S., Nyodu R., Maurya V.K., Saxena S.K. (2020). Morphology, genome organization, replication, and pathogenesis of severe acute respiratory syndrome coronavirus 2 (SARS-CoV-2). Coronavirus Disease 2019 (COVID-19): Epidemiology, Pathogenesis, Diagnosis, and Therapeutics.

[14] Le Bert N., Tan A., Kunasegaran K., Tham C., Hafezi M., Chia A., Chng M., Lin M., Tan N., Linster M. (2020). SARS-CoV-2-Specific T cell immunity in cases of COVID-19 and SARS, and uninfected controls. Nature.

[15] Li T., Wang L., Wang H., Li X., Wei W. (2020). Serum SARS-COV-2 nucleocapsid protein: A sensitivity and specificity early diagnostic marker for SARS-COV-2 infection. Front. Cell Infect. Microbiol..

[16] Li J., Lillehoj P.B. (2021). Microfluidic Magneto Immunosensor for Rapid, High sensitivity measurements of SARS-CoV-2 nucleocapsid protein in serum. ACS Sens..

[17] Yu M., Zhang X., Zhang X., Zahra Q.U.A., Huang Z., Chen Y., Song C., Song M., Jiang H., Luo Z. (2022). An electrochemical aptasensor with N protein binding aptamer-complementary oligonucleotide as probe for ultra-sensitive detection of COVID-19. Biosens. Bioelectron..

[18] Chen J., Tang J., Meng H., Liu Z., Wang L., Geng X., Wu Y., Qu L., Li Z. (2020). Recognition triggered assembly of split aptamers to initiate a hybridization chain reaction for wash-free and amplified detection of exosomes. Chem. Commun..

[19] Ahn D.G., Jeon I.J., Kim J., Song M.S., Han S.R. (2009). RNA aptamer-based sensitive detection of SARS coronavirus nucleocapsid protein. Analyst.

[20] Hong S.L., Xiang M.Q., Tang M., Pang D.W. (2019). Ebola virus aptamers: From highly efficient selection to application on magnetism-controlled chips. Anal. Chem..

[21] Bhardwaj J., Chaudhary N., Kim H., Jang J. (2019). Subtyping of influenza a H1N1 virus using a label-free electrochemical biosensor based on the DNA aptamer targeting the stem region of HA protein. Anal. Chim. Acta.

[22] Zhang L., Fang X., Liu X., Ou H., Luo Z. (2020). Discovery of the sandwich type COVID-19 nucleocapsid protein DNA aptamers. Chem. Commun..

[23] Liu P., Sun S., Guo X., Yang X., Huang J., Wang K., Wang Q., Liu J., He L. (2015). Competitive host-guest interaction between beta_cyclodextrin polymer and pyrene-labeled probes for fluorescence analyses. Anal. Chem..

[24] Gao S., Yang G., Zhang X., Lu Y., Chen Y., Wu X., Song C. (2022). β-Cyclodextrin polymer-based host–guest interaction and fluorescence enhancement of pyrene for sensitive isocarbophos detection. ACS Omega.

[25] Song C., Yang X., Wang K., Wang Q., Liu J., Huang J., He L., Liu P., Qing Z., Liu W. (2015). A Sensitive detection of T4 polynucleotide kinase activity based on beta-cyclodextrin polymer enhanced fluorescence combined with an exonuclease reaction. Chem. Commun..

[26] Song C., Xiao Y., Li K., Zhang X., Lu Y. (2019). Beta-cyclodextrin polymer based fluorescence enhancement method for sensitive adenosine triphosphate detection. Chin. Chem. Lett..

